# Di-μ_2_-acetato-di-μ_2_-azido-di-μ_3_-methanol-tetra­kis­{μ-2-[(2-methyl-1-oxidopropan-2-yl)imino­meth­yl]-6-meth­oxy­phenolato}tetra­nickel(II) methanol disolvate

**DOI:** 10.1107/S1600536811055164

**Published:** 2012-01-14

**Authors:** Shan-Yong Tan, Fei Chang, Yan-Peng Gao

**Affiliations:** a School of Chemistry and Chemical Engineering, Inner Mongolia University, Hohhot 010021, People’s Republic of China; b Department of Chemistry and Chemical Engineering, Ordos College of Inner Mongolia University, Erdos 017000, People’s Republic of China

## Abstract

In the centrosymmetric tetra­nuclear title complex, [Ni_4_(C_12_H_15_NO_3_)_2_(CH_3_COO)_2_(N_3_)_2_(CH_3_OH)_2_]·2CH_3_OH, the asymmetric unit comprises half of a complex mol­ecule and a methanol solvent mol­ecule. The Ni^II^ ions display two different coordination environments: (i) two O atoms from the Schiff base ligand, two O atoms from symmetry-related methanol mol­ecules and an O atom from an acetate group, one N atom from the azide group, and (ii) two O atoms and one N atom from the Schiff base, one O atom from methanol, one O atom from the acetate anion, and one N atom from the azide group. Four coplanar Ni^II^ ions are connected by two μ_2_-bridging O atoms from the two deprotonated Schiff bases, two μ_3_-O atoms from methanol mol­ecules, two μ_1,1_-N atoms from two azide ions, and four O atoms from acetate groups. The shortest Ni⋯Ni distance in the tetra­nuclear unit is 2.962 (2) Å. O—H⋯O hydrogen bonds between the methanol solvent mol­ecule and an acetate O atom feature in the crystal packing.

## Related literature

For applications of transition metal complexes with luminescent and magnetic properties, see: Pasatoiu, Sutter *et al.* (2011 ▶); Pasatoiu, Tiseanu *et al.* (2011 ▶); Sasmal, Hazra *et al.* (2011 ▶); Sasmal, Sarkar *et al.* (2011 ▶). For the preparation of the 2-[[(2-hy­droxy-1,1-dimethyl­eth­yl)imino]­meth­yl]-6-meth­oxy-phenol ligand, see: Rao *et al.* (1998 ▶). For related structures, see: Oshio *et al.* (2005 ▶); Nihei *et al.* (2007 ▶). 
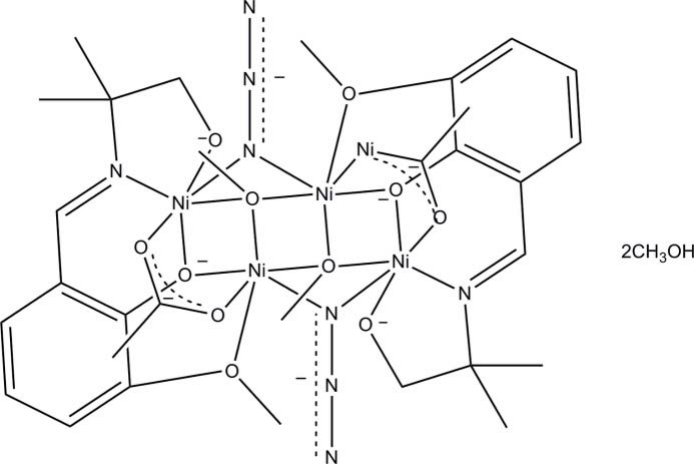


## Experimental

### 

#### Crystal data

[Ni_4_(C_12_H_15_NO_3_)_2_(C_2_H_3_O_2_)_2_(N_3_)_2_(CH_4_O)_2_]·2(CH_4_O)
*M* *_r_* = 1007.56
Monoclinic, 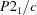

*a* = 9.5635 (14) Å
*b* = 11.8971 (16) Å
*c* = 18.845 (3) Å
β = 94.581 (2)°
*V* = 2137.3 (5) Å^3^
*Z* = 2
Mo *K*α radiation
μ = 1.81 mm^−1^
*T* = 296 K
0.2 × 0.2 × 0.2 mm

#### Data collection

Bruker APEXII CCD diffractometer
Absorption correction: multi-scan (*SADABS*; Bruker, 2004 ▶) *T*_min_ = 0.697, *T*_max_ = 0.704
15395 measured reflections
5277 independent reflections
4584 reflections with *I* > 2σ(*I*)
*R*_int_ = 0.017

#### Refinement

*R*[*F* ^2^ > 2σ(*F* ^2^)] = 0.035
*wR*(*F* ^2^) = 0.110
*S* = 0.84
5277 reflections
269 parameters
H-atom parameters constrained
Δρ_max_ = 1.47 e Å^−3^
Δρ_min_ = −0.55 e Å^−3^

### 

Data collection: *APEX2* (Bruker, 2004 ▶); cell refinement: *SAINT* (Bruker, 2004 ▶); data reduction: *SAINT*; program(s) used to solve structure: *SHELXS97* (Sheldrick, 2008 ▶); program(s) used to refine structure: *SHELXL97* (Sheldrick, 2008 ▶); molecular graphics: *SHELXTL* (Sheldrick, 2008 ▶); software used to prepare material for publication: *SHELXTL*.

## Supplementary Material

Crystal structure: contains datablock(s) I, global. DOI: 10.1107/S1600536811055164/kp2370sup1.cif

Structure factors: contains datablock(s) I. DOI: 10.1107/S1600536811055164/kp2370Isup2.hkl

Additional supplementary materials:  crystallographic information; 3D view; checkCIF report

## Figures and Tables

**Table 1:** Selected bond lengths (Å)

Ni1—O2	2.0052 (16)
Ni1—O3	2.0103 (18)
Ni1—O6^i^	2.0312 (17)
Ni1—N2	2.0720 (19)
Ni1—O6	2.0743 (16)
Ni1—O1	2.2897 (18)
Ni2—O2	1.9699 (16)
Ni2—N1	2.018 (2)
Ni2—O6	2.0282 (16)
Ni2—O5	2.0608 (18)
Ni2—O4	2.1709 (19)
Ni2—N2^i^	2.209 (2)

Symmetry code: (i) 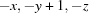.

**Table 2:** Hydrogen-bond geometry (Å, °)

*D*—H⋯*A*	*D*—H	H⋯*A*	*D*⋯*A*	*D*—H⋯*A*
O7—H7*A*⋯O4^ii^	0.82	1.88	2.698 (3)	175

Symmetry code: (ii) 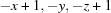.

**Fig. 1.**
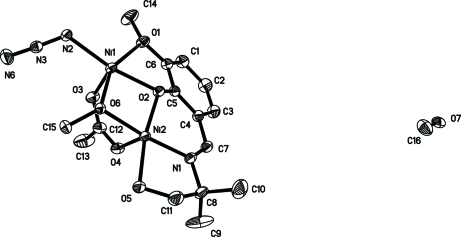
The asymmetric unit of the title compound with atom labels and 30% probability displacement ellipsoids. H ydrogen atoms are omitted for clarity.

**Fig. 2.**
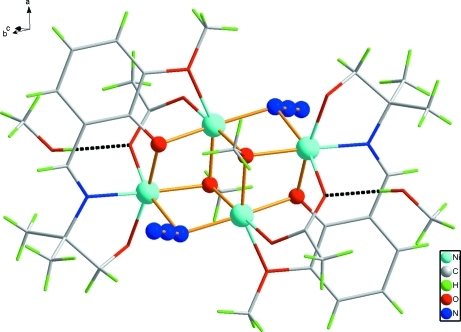
The structural unit is a tetranuclear complex which is generated from the asymmetric unit by an inversion symmetry operation -x, -y+1, -z. Hydrogen bonds are shown as dashed lines.

## supplementary crystallographic information

### Comment

Multidentate Schiff base ligands play an important role in assembly of metal coordination complexes. There is increasing interest in transition metal complexes with multidentate Schiff base ligands because of their potential applications in in luminescent and magnetic properties (Pasatoiu *et al.*, 2011; Sasmal, *et al.*, 2011). Herein, we report the synthesis and crystal structure of a tetranuclearnickel(II) complex (Fig. 1, Table 1) with a tridentate Schiff base ligand 2-[(3-methoxysalicylidene)amino]-2-methyl-1-propanol with the azide coligand. The tetranuclear complex is centrosymmetric and a half of the molecule comprises an asymmetric unit which reveals two different coordiantion environments; Ni1 is coordinated by two O atoms (O1 and O2) of the ligand, one O atom from acetato group (O3), two O atoms from methanol (O6 and O6^i^) and N from azido group (N2), whereas Ni2 is coordinated by the two O and one N atoms from the ligand (O2, O5,N1), one O atom from acetato group (O4), one O from methanol (O6), and N from azido group (N2)(Figs.1 and 2, Table 1). The solvent methanol molecule acts as a proton donor to acetato group (O4) (Table 2, Fig. 2).

### Experimental

2-[(3-Methoxysalicylidene)amino]-2-methyl-1-propanol (0.1 mmol) in 15 mL of methyl alcohol is stirred for 30 m at room temperature. Then triethylamine(0.2 mmol) was added and stirred. After 10 m nickel(II) acetate tetrahydrate (0.15 mmol), sodium azide (0.1 mmol) were added and stirred for another 10 m. The resulting solution was filtered and left to evaporate slowly at room temperature for 4 days, giving green block crystals of the title complex for X-ray structure analysis.

### Refinement

The H atom of the coordinating methanol molecule could not be located and was excluded from the model. The other H atoms were placed at geometrically idealised positions (C—H = 0.93–0.97Å and O—H = 0.82 Å), with *U*_iso_(H) = 1.2*U*_eq_(C) and *U*_iso_(H) = 1.5*U*_eq_(O).

### Crystal data

**Table:**

[Ni_4_(C_12_H_15_NO_3_)_2_(C_2_H_3_O_2_)_2_(N_3_)_2_(CH_4_O)_2_]·2(CH_4_O)	*F*(000) = 1044.0
*M**_r_* = 1007.56	*D*_x_ = 1.563 Mg m^−^^3^
Monoclinic, *P*2_1_/*c*	Mo *K*α radiation, λ = 0.71073 Å
Hall symbol: -P 2ybc	Cell parameters from 8404 reflections
*a* = 9.5635 (14) Å	θ = 2.7–28.2°
*b* = 11.8971 (16) Å	µ = 1.81 mm^−^^1^
*c* = 18.845 (3) Å	*T* = 296 K
β = 94.581 (2)°	Block, green
*V* = 2137.3 (5) Å^3^	0.2 × 0.2 × 0.2 mm
*Z* = 2	

### Data collection

**Table:**

Bruker APEXII CCD diffractometer	5277 independent reflections
Radiation source: sealed tube	4584 reflections with *I* > 2σ(*I*)
graphite	*R*_int_ = 0.017
φ and ω scans	θ_max_ = 28.3°, θ_min_ = 2.0°
Absorption correction: multi-scan (*SADABS*; Bruker, 2004)	*h* = −12→12
*T*_min_ = 0.697, *T*_max_ = 0.704	*k* = −13→15
15395 measured reflections	*l* = −25→23

### Refinement

**Table:**

Refinement on *F*^2^	Primary atom site location: structure-invariant direct methods
Least-squares matrix: full	Secondary atom site location: difference Fourier map
*R*[*F*^2^ > 2σ(*F*^2^)] = 0.035	Hydrogen site location: inferred from neighbouring sites
*wR*(*F*^2^) = 0.110	H-atom parameters constrained
*S* = 0.84	*w* = 1/[σ^2^(*F*_o_^2^) + (0.0832*P*)^2^ + 2.6493*P*] where *P* = (*F*_o_^2^ + 2*F*_c_^2^)/3
5277 reflections	(Δ/σ)_max_ = 0.001
269 parameters	Δρ_max_ = 1.47 e Å^−^^3^
0 restraints	Δρ_min_ = −0.55 e Å^−^^3^

### Special details

**Table:**

Geometry. All e.s.d.'s (except the e.s.d. in the dihedral angle between two l.s. planes) are estimated using the full covariance matrix. The cell e.s.d.'s are taken into account individually in the estimation of e.s.d.'s in distances, angles and torsion angles; correlations between e.s.d.'s in cell parameters are only used when they are defined by crystal symmetry. An approximate (isotropic) treatment of cell e.s.d.'s is used for estimating e.s.d.'s involving l.s. planes.
Refinement. Refinement of *F*^2^ against ALL reflections. The weighted *R*-factor *wR* and goodness of fit *S* are based on *F*^2^, conventional *R*-factors *R* are based on *F*, with *F* set to zero for negative *F*^2^. The threshold expression of *F*^2^ > σ(*F*^2^) is used only for calculating *R*-factors(gt) *etc*. and is not relevant to the choice of reflections for refinement. *R*-factors based on *F*^2^ are statistically about twice as large as those based on *F*, and *R*- factors based on ALL data will be even larger.

### Fractional atomic coordinates and isotropic or equivalent isotropic displacement parameters (Å^2^)

**Table:**

	*x*	*y*	*z*	*U*_iso_*/*U*_eq_	
Ni1	0.15407 (3)	0.47649 (2)	0.012265 (15)	0.02862 (10)	
Ni2	−0.03379 (3)	0.29667 (2)	0.057375 (15)	0.02882 (10)	
C6	0.3281 (2)	0.4368 (2)	0.15200 (13)	0.0337 (5)	
C5	0.2010 (2)	0.37608 (19)	0.15074 (12)	0.0299 (4)	
C7	0.0623 (3)	0.2180 (2)	0.20063 (14)	0.0388 (5)	
H7	0.0544	0.1718	0.2399	0.047*	
C8	−0.1572 (3)	0.1358 (2)	0.15586 (15)	0.0447 (6)	
C3	0.2992 (3)	0.2680 (2)	0.25272 (14)	0.0436 (6)	
H3	0.2901	0.2130	0.2871	0.052*	
C4	0.1870 (3)	0.2889 (2)	0.20048 (13)	0.0337 (5)	
C1	0.4383 (3)	0.4150 (2)	0.20212 (15)	0.0428 (6)	
H1	0.5213	0.4559	0.2026	0.051*	
C2	0.4218 (3)	0.3292 (3)	0.25254 (16)	0.0494 (7)	
H2	0.4952	0.3135	0.2864	0.059*	
O2	0.10001 (16)	0.40585 (14)	0.10273 (8)	0.0309 (3)	
O1	0.32663 (18)	0.51793 (15)	0.09962 (10)	0.0396 (4)	
N1	−0.0370 (2)	0.21444 (17)	0.15087 (11)	0.0352 (4)	
C11	−0.2605 (4)	0.1602 (3)	0.08853 (18)	0.0558 (8)	
H11A	−0.3239	0.0971	0.0801	0.067*	
H11B	−0.3161	0.2263	0.0970	0.067*	
C9	−0.1127 (5)	0.0167 (3)	0.1622 (4)	0.1006 (19)	
H9A	−0.0507	−0.0003	0.1261	0.151*	
H9B	−0.1937	−0.0311	0.1564	0.151*	
H9C	−0.0650	0.0042	0.2083	0.151*	
C10	−0.2433 (4)	0.1707 (5)	0.2188 (2)	0.0819 (13)	
H10A	−0.3341	0.1360	0.2133	0.123*	
H10B	−0.2538	0.2510	0.2191	0.123*	
H10C	−0.1951	0.1468	0.2628	0.123*	
C12	0.2533 (3)	0.2440 (2)	0.00123 (15)	0.0405 (5)	
C13	0.3687 (4)	0.1640 (3)	−0.0181 (3)	0.0784 (13)	
H13A	0.3638	0.1544	−0.0688	0.118*	
H13B	0.3565	0.0925	0.0042	0.118*	
H13C	0.4585	0.1946	−0.0019	0.118*	
O4	0.14558 (19)	0.20252 (15)	0.02585 (11)	0.0402 (4)	
O3	0.27595 (19)	0.34597 (15)	−0.01089 (11)	0.0412 (4)	
C14	0.4556 (3)	0.5730 (4)	0.0879 (2)	0.0688 (11)	
H14A	0.4880	0.6143	0.1298	0.103*	
H14B	0.4409	0.6237	0.0484	0.103*	
H14C	0.5246	0.5179	0.0777	0.103*	
O5	−0.1839 (2)	0.17807 (17)	0.02701 (10)	0.0444 (4)	
O6	−0.02391 (17)	0.39361 (13)	−0.03062 (8)	0.0297 (3)	
C15	−0.0307 (3)	0.3404 (2)	−0.09846 (13)	0.0413 (6)	
H15A	0.0460	0.2886	−0.1001	0.062*	
H15B	−0.0248	0.3962	−0.1349	0.062*	
H15C	−0.1178	0.3005	−0.1062	0.062*	
N2	0.1997 (2)	0.57829 (17)	−0.07205 (10)	0.0321 (4)	
N3	0.2050 (2)	0.54193 (18)	−0.13116 (12)	0.0358 (4)	
N6	0.2121 (3)	0.5107 (3)	−0.18836 (15)	0.0584 (7)	
O7	0.8278 (3)	0.0154 (2)	0.93586 (16)	0.0720 (7)	
H7A	0.8392	−0.0513	0.9455	0.108*	
C16	0.7350 (7)	0.0260 (4)	0.8735 (3)	0.0965 (16)	
H16A	0.6900	0.0982	0.8733	0.145*	
H16B	0.6653	−0.0321	0.8729	0.145*	
H16C	0.7870	0.0192	0.8322	0.145*	

### Atomic displacement parameters (Å^2^)

**Table:**

	*U*^11^	*U*^22^	*U*^33^	*U*^12^	*U*^13^	*U*^23^
Ni1	0.02667 (16)	0.02808 (16)	0.03090 (16)	−0.00306 (10)	0.00100 (11)	0.00428 (10)
Ni2	0.03015 (16)	0.02786 (16)	0.02811 (16)	−0.00462 (10)	0.00031 (11)	0.00272 (10)
C6	0.0309 (11)	0.0338 (11)	0.0360 (11)	−0.0005 (9)	−0.0006 (9)	0.0017 (9)
C5	0.0304 (10)	0.0299 (10)	0.0287 (10)	−0.0007 (8)	−0.0018 (8)	0.0006 (8)
C7	0.0458 (14)	0.0361 (12)	0.0340 (12)	−0.0069 (10)	0.0000 (10)	0.0060 (9)
C8	0.0467 (14)	0.0461 (14)	0.0408 (13)	−0.0186 (12)	0.0000 (11)	0.0120 (11)
C3	0.0437 (14)	0.0452 (14)	0.0402 (13)	0.0021 (11)	−0.0079 (11)	0.0083 (11)
C4	0.0359 (12)	0.0344 (11)	0.0298 (11)	−0.0017 (9)	−0.0033 (9)	0.0044 (9)
C1	0.0321 (12)	0.0489 (15)	0.0458 (14)	−0.0027 (10)	−0.0066 (10)	0.0009 (11)
C2	0.0413 (14)	0.0584 (17)	0.0457 (15)	−0.0002 (13)	−0.0132 (11)	0.0060 (13)
O2	0.0292 (7)	0.0322 (8)	0.0302 (7)	−0.0057 (6)	−0.0039 (6)	0.0051 (6)
O1	0.0320 (8)	0.0405 (9)	0.0453 (10)	−0.0107 (7)	−0.0027 (7)	0.0085 (7)
N1	0.0394 (11)	0.0333 (10)	0.0328 (10)	−0.0077 (8)	0.0027 (8)	0.0050 (8)
C11	0.0539 (17)	0.0601 (19)	0.0527 (17)	−0.0169 (15)	−0.0008 (13)	0.0054 (14)
C9	0.068 (3)	0.0384 (18)	0.192 (6)	−0.0131 (17)	−0.010 (3)	0.030 (3)
C10	0.061 (2)	0.117 (4)	0.070 (2)	−0.028 (2)	0.0204 (19)	0.000 (2)
C12	0.0384 (12)	0.0334 (12)	0.0500 (14)	−0.0006 (10)	0.0048 (10)	−0.0017 (10)
C13	0.056 (2)	0.0417 (17)	0.143 (4)	0.0086 (15)	0.040 (2)	−0.002 (2)
O4	0.0397 (10)	0.0308 (9)	0.0506 (11)	−0.0015 (7)	0.0063 (8)	0.0009 (7)
O3	0.0385 (9)	0.0331 (9)	0.0533 (11)	0.0002 (7)	0.0114 (8)	0.0040 (8)
C14	0.0458 (17)	0.081 (3)	0.078 (2)	−0.0329 (17)	−0.0057 (15)	0.028 (2)
O5	0.0511 (11)	0.0456 (10)	0.0362 (9)	−0.0194 (9)	0.0015 (8)	−0.0011 (8)
O6	0.0330 (8)	0.0305 (8)	0.0254 (7)	−0.0029 (6)	0.0002 (6)	0.0011 (6)
C15	0.0497 (14)	0.0438 (14)	0.0304 (11)	−0.0052 (11)	0.0024 (10)	−0.0068 (10)
N2	0.0319 (9)	0.0334 (10)	0.0315 (9)	−0.0026 (7)	0.0047 (7)	0.0037 (8)
N3	0.0319 (10)	0.0362 (10)	0.0397 (11)	−0.0032 (8)	0.0051 (8)	0.0011 (8)
N6	0.0670 (18)	0.0652 (17)	0.0447 (14)	−0.0091 (14)	0.0151 (12)	−0.0128 (12)
O7	0.103 (2)	0.0377 (12)	0.0759 (17)	0.0026 (13)	0.0132 (15)	−0.0054 (11)
C16	0.136 (5)	0.079 (3)	0.071 (3)	0.019 (3)	−0.007 (3)	−0.006 (2)

### Geometric parameters (Å, °)

**Table:**

Ni1—O2	2.0052 (16)	C11—O5	1.436 (4)
Ni1—O3	2.0103 (18)	C11—H11A	0.9700
Ni1—O6^i^	2.0312 (17)	C11—H11B	0.9700
Ni1—N2	2.0720 (19)	C9—H9A	0.9600
Ni1—O6	2.0743 (16)	C9—H9B	0.9600
Ni1—O1	2.2897 (18)	C9—H9C	0.9600
Ni1—Ni1^i^	2.9988 (7)	C10—H10A	0.9600
Ni2—O2	1.9699 (16)	C10—H10B	0.9600
Ni2—N1	2.018 (2)	C10—H10C	0.9600
Ni2—O6	2.0282 (16)	C12—O3	1.256 (3)
Ni2—O5	2.0608 (18)	C12—O4	1.263 (3)
Ni2—O4	2.1709 (19)	C12—C13	1.524 (4)
Ni2—N2^i^	2.209 (2)	C13—H13A	0.9600
C6—O1	1.380 (3)	C13—H13B	0.9600
C6—C1	1.382 (3)	C13—H13C	0.9600
C6—C5	1.413 (3)	C14—H14A	0.9600
C5—O2	1.318 (3)	C14—H14B	0.9600
C5—C4	1.411 (3)	C14—H14C	0.9600
C7—N1	1.281 (3)	O6—C15	1.424 (3)
C7—C4	1.461 (3)	O6—Ni1^i^	2.0313 (17)
C7—H7	0.9300	C15—H15A	0.9600
C8—C9	1.481 (5)	C15—H15B	0.9600
C8—N1	1.491 (3)	C15—H15C	0.9600
C8—C10	1.553 (5)	N2—N3	1.200 (3)
C8—C11	1.572 (4)	N2—Ni2^i^	2.209 (2)
C3—C2	1.380 (4)	N3—N6	1.147 (3)
C3—C4	1.419 (3)	O7—C16	1.421 (6)
C3—H3	0.9300	O7—H7A	0.8200
C1—C2	1.412 (4)	C16—H16A	0.9600
C1—H1	0.9300	C16—H16B	0.9600
C2—H2	0.9300	C16—H16C	0.9600
O1—C14	1.429 (3)		
			
O2—Ni1—O3	93.10 (7)	C6—O1—C14	118.1 (2)
O2—Ni1—O6^i^	88.34 (7)	C6—O1—Ni1	109.24 (13)
O3—Ni1—O6^i^	176.78 (7)	C14—O1—Ni1	124.70 (19)
O2—Ni1—N2	168.94 (7)	C7—N1—C8	120.3 (2)
O3—Ni1—N2	97.11 (8)	C7—N1—Ni2	124.16 (17)
O6^i^—Ni1—N2	81.68 (7)	C8—N1—Ni2	115.14 (16)
O2—Ni1—O6	82.69 (6)	O5—C11—C8	110.5 (3)
O3—Ni1—O6	91.16 (7)	O5—C11—H11A	109.5
O6^i^—Ni1—O6	86.16 (7)	C8—C11—H11A	109.5
N2—Ni1—O6	101.29 (7)	O5—C11—H11B	109.5
O2—Ni1—O1	72.48 (6)	C8—C11—H11B	109.5
O3—Ni1—O1	85.71 (8)	H11A—C11—H11B	108.1
O6^i^—Ni1—O1	97.47 (7)	C8—C9—H9A	109.5
N2—Ni1—O1	103.98 (7)	C8—C9—H9B	109.5
O6—Ni1—O1	154.73 (6)	H9A—C9—H9B	109.5
O2—Ni1—Ni1^i^	83.82 (5)	C8—C9—H9C	109.5
O3—Ni1—Ni1^i^	133.65 (6)	H9A—C9—H9C	109.5
O6^i^—Ni1—Ni1^i^	43.64 (4)	H9B—C9—H9C	109.5
N2—Ni1—Ni1^i^	92.14 (6)	C8—C10—H10A	109.5
O6—Ni1—Ni1^i^	42.52 (5)	C8—C10—H10B	109.5
O1—Ni1—Ni1^i^	135.53 (5)	H10A—C10—H10B	109.5
O2—Ni2—N1	89.73 (7)	C8—C10—H10C	109.5
O2—Ni2—O6	84.76 (6)	H10A—C10—H10C	109.5
N1—Ni2—O6	174.03 (7)	H10B—C10—H10C	109.5
O2—Ni2—O5	170.33 (7)	O3—C12—O4	127.0 (2)
N1—Ni2—O5	81.47 (8)	O3—C12—C13	114.9 (2)
O6—Ni2—O5	103.84 (7)	O4—C12—C13	118.1 (3)
O2—Ni2—O4	87.64 (7)	C12—C13—H13A	109.5
N1—Ni2—O4	93.21 (8)	C12—C13—H13B	109.5
O6—Ni2—O4	88.87 (7)	H13A—C13—H13B	109.5
O5—Ni2—O4	96.82 (8)	C12—C13—H13C	109.5
O2—Ni2—N2^i^	87.13 (7)	H13A—C13—H13C	109.5
N1—Ni2—N2^i^	98.98 (8)	H13B—C13—H13C	109.5
O6—Ni2—N2^i^	78.48 (7)	C12—O4—Ni2	125.71 (17)
O5—Ni2—N2^i^	90.26 (8)	C12—O3—Ni1	126.49 (17)
O4—Ni2—N2^i^	166.70 (7)	O1—C14—H14A	109.5
O1—C6—C1	125.7 (2)	O1—C14—H14B	109.5
O1—C6—C5	112.69 (19)	H14A—C14—H14B	109.5
C1—C6—C5	121.6 (2)	O1—C14—H14C	109.5
O2—C5—C4	123.4 (2)	H14A—C14—H14C	109.5
O2—C5—C6	117.2 (2)	H14B—C14—H14C	109.5
C4—C5—C6	119.4 (2)	C11—O5—Ni2	105.45 (16)
N1—C7—C4	125.1 (2)	C15—O6—Ni2	118.66 (15)
N1—C7—H7	117.5	C15—O6—Ni1^i^	120.45 (15)
C4—C7—H7	117.5	Ni2—O6—Ni1^i^	102.96 (7)
C9—C8—N1	112.8 (3)	C15—O6—Ni1	122.57 (15)
C9—C8—C10	111.1 (4)	Ni2—O6—Ni1	92.42 (6)
N1—C8—C10	109.8 (3)	Ni1^i^—O6—Ni1	93.84 (7)
C9—C8—C11	113.6 (3)	O6—C15—H15A	109.5
N1—C8—C11	105.7 (2)	O6—C15—H15B	109.5
C10—C8—C11	103.3 (3)	H15A—C15—H15B	109.5
C2—C3—C4	120.1 (3)	O6—C15—H15C	109.5
C2—C3—H3	120.0	H15A—C15—H15C	109.5
C4—C3—H3	120.0	H15B—C15—H15C	109.5
C5—C4—C3	118.9 (2)	N3—N2—Ni1	121.89 (17)
C5—C4—C7	123.1 (2)	N3—N2—Ni2^i^	116.32 (16)
C3—C4—C7	118.0 (2)	Ni1—N2—Ni2^i^	95.75 (8)
C6—C1—C2	118.4 (2)	N6—N3—N2	177.6 (3)
C6—C1—H1	120.8	C16—O7—H7A	109.5
C2—C1—H1	120.8	O7—C16—H16A	109.5
C3—C2—C1	121.6 (2)	O7—C16—H16B	109.5
C3—C2—H2	119.2	H16A—C16—H16B	109.5
C1—C2—H2	119.2	O7—C16—H16C	109.5
C5—O2—Ni2	122.38 (14)	H16A—C16—H16C	109.5
C5—O2—Ni1	118.15 (14)	H16B—C16—H16C	109.5
Ni2—O2—Ni1	96.32 (7)		

Symmetry codes: (i) −*x*, −*y*+1, −*z*.

### Hydrogen-bond geometry (Å, °)

**Table:**

*D*—H···*A*	*D*—H	H···*A*	*D*···*A*	*D*—H···*A*
O7—H7A···O4^ii^	0.82	1.88	2.698 (3)	175

Symmetry codes: (ii) −*x*+1, −*y*, −*z*+1.
